# Motivations to Enhance One’s Facial Hair: Affiliation, Rivalry, and Stress

**DOI:** 10.1007/s10508-024-02919-0

**Published:** 2024-06-17

**Authors:** Marcin Moroń, Łukasz Jach, Peter K. Jonason

**Affiliations:** 1 Institute of Psychology, University of Silesia in Katowice, Grażyńskiego Street 53, 40-126 Katowice, Poland; 2 Department of General Psychology, University of Padua, Padua, Italy; 3 Cross-Cultural Psychology Centre, Cardinal Stefan Wyszyński University, Warsaw, Poland

**Keywords:** Facial hair, Fundamental social motives, Gender role stress, Intrasexual competition

## Abstract

Facial hair is a sexually dimorphic trait with potential evolutionary and sociocultural functions. Bearded men are perceived as dominant, aggressive, and masculine, but also as having better parenting skills. Men may intentionally manage the amount and shape of their facial hair as a part of their self-promotion strategy; however, facial hair management entails costs in terms of time, effort, and money. We explored psychological factors associated with facial hair enhancement motivation among men. A total of 414 men (aged 18–40 years) reported the current amount of their facial hair along with their facial hair enhancement motivations, gender role stress, intrasexual competitiveness, and fundamental social motives. Willingness to care for facial hair was associated with the amount of facial hair men claimed to have and correlated with the fundamental social motives of affiliation and social status, intrasexual competition, and gender role stress. Therefore, facial hair enhancement may be regarded as a self-presentation strategy aimed at acquiring a beneficial position in social networks.

**Supplementary Information:**

The online version contains supplementary material available at 10.1007/s10508-024-02919-0.

## Introduction

Beards and various facial hair styles (e.g., goatees, mustaches, etc.) are popular in many cultures, especially in large cities (Dixson et al., 2017; Gray et al., 2020). In humans, facial hair is a sexually dimorphic feature that appears in men during puberty and is driven by the action of androgens (Randall, 1994, 2008). The amount of facial hair varies markedly between men and women, raising questions about its intersexual (Darwin, 1871) and intrasexual (Guthrie, 1970; Muscarella & Cunningham, 1996) functions. Moreover, it could be a conspicuous ornamentation (i.e. beardedness); men are like male nonhuman primates with polygynous mating systems (Dixson et al., 2005) and large social group sizes with multilevel social organization (Grueter et al., 2015). These conditions favor sexually selected ornaments that signal age, social status, and dominance involved primarily in male-male competition and secondarily in attractiveness to females. Most research conducted so far focused on the social perceptions of men with different amounts of facial hair with a small part of the research concerning men’s preferences related to facial hair (Jach & Moroń, 2020; Jach et al., 2023). From this perspective, men may intentionally manage the amount and shape of their facial hair as a part of their self-promotion strategy (Davis & Arnocky, 2022a) by enhancing the visibility of their facial hair, removing facial hair, or giving it a specific shape. However, facial hair management entails costs in terms of time, effort, and money which is reflected in the growing male cosmetics business that men may be unequally willing to expend (Davis & Arnocky, 2022c). Therefore, our goal was to explore the psychological factors associated with facial hair enhancement motivation among men. We focused on factors related to men’s functioning in contexts involving their sex and gender. Thus, we studied the associations between men’s facial hair enhancement motivation with fundamental social motives, intrasexual competition, and gender role stress.

People link facial hair with biological characteristics such as sexual maturity, age, and masculinity (Dixson & Vasey, 2012; Gray et al., 2020); however, not only biological factors but also socio-psychological factors regulate their functioning. People live in large social groups that have long-term stability and coherence, consisting of both related and unrelated individuals (Sutcliffe et al., 2012) and distinct components of a human social system are social organization, social structure, mating system, and care system (Kappeler, 2019). Psychological reflections of these social conditions are fundamental social motives that are systems shaped by humans’ evolutionary history to energize, organize, and select behaviors to manage recurrent social threats and reproductive opportunities (Neel et al., 2016). The list of people’s fundamental social motives includes self-protection, disease avoidance, affiliation to a group, to friends, and to avoid peer exclusion, status-seeking, mate-seeking, mate retention, kin care related to family members, and kin care related to children (Ko et al., 2020).

Studies conducted so far have shown that people give different characteristics related to fundamental social motives to men with different types of facial hair. However, research on perceptions of men's facial hair provides mixed findings and there is a need for more research on the topic to help clarify this ambiguity. For example, people rate bearded men as healthier (Dixson & Brooks, 2013) and having better fighting abilities (Třebický et al., 2019) which may be related to the motives of disease avoidance and self-protection. Bearded men also are perceived as more trustworthy (Bakmazian, 2014) and friendly (Craig et al., 2019), which may correspond to affiliative social motives. On the other hand, men with facial hair are perceived as more aggressive, more dominant, and having more social status (Dixson & Vasey, 2012; Mefodeva et al., 2020), which may translate into a status-seeking motive. In the context of mate-seeking and mate-retention motives, facial hair modifies the assessment of men’s attractiveness (Clarkson et al., 2020; Stower et al., 2020); however, the results related to this issue are ambiguous (Jach & Moroń, 2020). Lastly, people attribute greater fathering abilities (Dixson et al., 2019) to men with facial hair, which may refer to familial care-related social motives.

We hypothesized that men’s striving to realize fundamental social motives might manifest through increased interest in facial hair enhancement; therefore, we predicted positive correlations between facial hair enhancement motivation and orientation on such fundamental social motives as self-protection, status-seeking, mate-seeking, and kin care related to children.

The social environment typical for human groups stimulates mechanisms of intrasexual competition defined as the degree to which one views the confrontation with same-sex individuals, especially in the context of contact with the opposite sex, in competitive terms (Buunk & Fisher, 2009; Dixson et al., 2017). Men compete with other men for partners as well as material and social resources to acquire and keep partners (Arnocky & Carré, 2016). Previous research showed that facial hair can play a role in both direct and indirect competition between men. First, facial hair may protect men in physical contests (Beseris et al., 2020, but see Třebický et al., 2019) or may discourage other men from competing by presenting its wearer as physically stronger (Fink et al., 2007) and more aggressive (Dixson & Vasey, 2012). Moreover, masculine craniofacial features overlap with the musculature employed when posing facial displays of anger, and beards enhance the speed and accuracy of detecting angry facial expressions compared to clean-shaven faces (Craig et al., 2019; Dixson et al., 2021). Second, because bearded men are perceived as older and with more social status (Dixson & Vasey, 2012; Mefodeva et al., 2020) other men may be more motivated to build coalitions with bearded men rather than come into conflict with them. Moreover, beards augment perceptions of masculinity, dominance, and aggressiveness in feminine faces with beards over masculine clean-shaven faces, possibly by enhancing masculine features or masking feminine features (Mefodeva et al., 2020). Because facial hair modifies the social perception of physical and symbolic traits involved in men’s intrasexual competition we hypothesized that men with a greater tendency to intrasexual competition would be more motivated to enhance their facial hair as a way of impressing or intimidating other men. Thus, we predicted positive correlations between facial hair enhancement motivation and dimensions of intrasexual competition: envy (i.e., wanting what others have), jealousy (i.e., protecting what one has), and superiority (i.e., beliefs that one is better; Jonason et al., 2023).

According to the theory of precarious manhood, men’s self-esteem is largely dependent on social signals indicating that they fulfill their roles and expectations well (Vandello et al., 2008). Men experience masculine gender role stress when they feel they are not meeting society’s expectations for masculine behavior or when the situation forces men to act in feminine-typed ways (Arrindell et al., 2013; Eisler & Skidmore, 1987). Beardedness is associated with perceived masculinity of a man (Addison, 1989; Mefodeva et al., 2020); moreover, studies on gender schemas showed that physical appearance plays a dominant role among other components of gender stereotypes (Deaux & Lewis, 1984). Therefore, facial hair could be regarded as an important component of masculine gender roles like muscle size or physique (Jackson et al., 1987; McCabe & Riciardelli, 2004). Bearded men report feeling more masculine (Wood, 1986); therefore, growing and displaying facial hair could help to establish and maintain higher self-perceived masculinity. Because wearing facial hair may be considered as a socially constructed manifestation of masculinity (Addison, 1989; Mefodeva et al., 2020) we hypothesized that men’s facial hair enhancement motivation may be related to their need to express masculinity in various social contexts. Therefore, we predicted positive correlations between facial hair enhancement motivation and gender role stress dimensions of our participants.

In our study, we decided to control the actual facial hair amount and age of participants. Facial hair enhancement motivation can be derived from their actual facial hair because more facial hair requires more effort to keep it clean; from another perspective, also maintaining a clean-shaven face requires the effort of frequent facial hair removal. From another perspective, regular care of facial hair requires time, effort, and resources. Thus, facial hair enhancement motivation may be related to the desire to present oneself to other people as a resourceful and well-organized person (cf. Miller, 2009), regardless of the actual length of facial hair. Additionally, people perceive bearded men as older (Dixson & Vasey, 2012) suggesting that men’s facial hair management behavior may be related to their motivation to appear younger or older compared to their age.

## Method

### Participants and Procedure

A sample of 414 heterosexual men from Poland who were aged 18–40 years (*M* = 29.13, *SD* = 6.65) consented to participate in an anonymous, online study via the SW Research survey platform in exchange for points that could be exchanged for prizes. The participants were informed of the nature of the study and if they consented via tick-box, they provided information about their facial hair and completed questionnaires measuring facial hair enhancement motivation, fundamental social motives, gender role stress, and intrasexual competition. Upon completion, they were thanked, debriefed, and remunerated. Sensitivity analysis indicated that the sample size was large enough to detect small correlations (|*r*|= 0.14), small effect size in two independent groups comparison (Cohen’s *d* = 0.24), and small effect size in four independent groups comparison (*f* = 0.16) with appropriate power (1-β = 0.80) given α equal to 0.05 (Faul et al., 2007).

### Measures

**Fig. 1:**
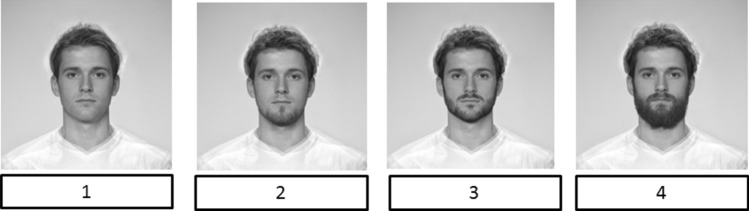
Morphed faces showing different types of facial hair. *Note*: 1 = “Clean-shaven”, 2 = “Light stubble”, 3 = “Heavy stubble”, 4 = “Full beard”

We used four specially designed photos of faces with different types of facial hair to measure actual facial hair which the participants had when they participated in the study. We prepared these photos using the FaceApp application, and our primary face was a morphed, average face of a white young adult clean-shaven male provided by DeBruine (2016). Next, we added three increasing levels of facial hair: light stubble, heavy stubble, and full beard, presented in Fig. 1. Participants indicated which photo best reflected their facial hair.

We measured facial hair enhancement motivation with three items. Participants reported how likely (1 = *Not at all*; 7 = *Definitely yes*) they would be willing to use professional beard and facial hair services, buy facial hair care products, and spend time trimming and styling their facial hair. The items were averaged to create an index of the propensity to engage in facial hair enhancement (Cronbach’s α = 0.89; *M* = 4.15, *SD* = 1.61).

We measured individual differences in the fundamental social motives with single-item scales. We asked participants to rate the importance of such motives as self-protection, disease avoidance, affiliation to a group, affiliation to friends, affiliation related to exclusion concern, status-seeking, mate-seeking, mate retention, kin care related to family members, and kin care related to children (Ko et al., 2020). Participants read a brief description of each life goal (e.g., “Staying safe from dangerous people” related to self-protection) and rated their importance (1 = *Definitely unimportant*; 7 = *Definitely important*).

To measure the level of gender role stress experienced by the respondents, we translated the 15-item Masculine Gender Role Stress Scale (Arrindell et al., 2013) into Polish. This tool consists of five subscales (3-item each) that describe situations related to physical inadequacy (e.g., “Not being able to find a sexual partner”; α = 0.70), emotional inexpressiveness (e.g., “Telling your partner that you love her”; α = 0.71), subordination to women (e.g., “Being with a woman who is more successful than you”; α = 0.87), intellectual inferiority (e.g., “Working with people who seem more ambitious than you”; α = 0.85), and performance failure (e.g., “Being unable to perform sexually”; α = 0.74). Participants rated how stressful the situations described in the items were for them (1 = *Not at all*; 7 = *Extremely*). Items were averaged to create indexes of each.

We measured individual differences in intrasexual competition with our own translation of the 12-item Intrasexual Competition Scale (Buunk & Fisher, 2009) reduced to its three dimensions (Jonason et al., 2023) of envy (4 items; e.g., “I can’t stand it when I meet another man who is more attractive than I am”; α = 0.83), jealousy (4 items; e.g., “I tend to look for negative characteristics in men who are very successful.”; α = 0.88), and superiority (4 items; e.g., “I always want to beat other men/women”; α = 0.79). Participants reported how applicable (1 = *Not at all*; 7 = *Completely*) each item was to them. Items were averaged to create indexes of each dimension of intrasexual competition. We also calculated the average score related to overall intrasexual competition (α = 0.90).

### Analysis Plan

First, we examined descriptive statistics for all studied variables. Then, we analyzed the associations between the participants’ facial hair amount and fundamental social motives, gender role stress, and intrasexual competition using Pearson correlations. Next, we investigated the association between motivation to enhance one’s facial hair and fundamental social motives, gender role stress, and intrasexual competition using Pearson correlations and partial correlations (controlled for age and actual facial hair). We also used hierarchical regression analysis to check whether the associations between motivation to enhance one’s facial hair and other studied variables were moderated by the level of facial hair. In each regression model, we partialed the variance shared with other dimensions of the analyzed construct. Last, we investigated differences in the strength of the associations between fundamental social motives, gender role stress, intrasexual rivalry, and facial hair enhancement motives. We used Steiger’s (1980) *z* test for differences between dependent correlation coefficients. To reduce the false discovery rate, we applied the Benjamini and Hochberg (1995) correction.

## Results

We reported means and standard deviations of fundamental social motives, gender role stress, and intrasexual competition in Table 1. From among the facial hair types presented, participants chose the one that resembles their facial hair amount: clean-shaven (*n* = 202), light stubble (*n* = 86), heavy stubble (*n* = 102), and full beard (*n* = 24). We observed correlations between facial hair enhancement motivation and facial hair amount (*r* = 0.25; *p* < 0.001), facial hair amount and age (*r* = 0.12; *p* = 0.01), and facial hair enhancement motivation and age (*r* = − 0.12; *p* = 0.01); therefore, we calculated partial correlations between facial hair enhancement motivation and fundamental social motives, gender role stress, and intrasexual competition controlling for facial hair amount and age (Table 1; correlations between fundamental social motives, gender role stress, and intrasexual rivalry are given in Table S1 in Supplementary online material). Older men reported higher motivation related to self-protection, disease avoidance, mate retention, and kin care related to children. We did not find correlations between age and gender role stress, and intrasexual rivalry. Facial hair amount did not correlate with fundamental social motives, gender role stress, and intrasexual competition. However, men with more facial hair enhancement motivation reported higher motivation in eight out of ten fundamental social motives, were more competitive in all dimensions of intrasexual rivalry, and reported higher role distress in four out of five dimensions of gender role stress. Correlations between facial hair enhancement motivation and other variables remained stable after controlling facial hair amount and age, and after controlling for the false discovery rate.

**Table 1:** Descriptive statistics and correlations between participants’ facial hair and facial hair enhancement motivations and fundamental social motives, gender role stress, and intrasexual competition

Variable	Mean (*SD*)	Age	Facial hair amount	Facial hair enhancement motivation
*Fundamental social motives*				
Self-protection	5.31 (1.31)	.18**/**	.11*/*ns*	.08
Disease avoidance	5.73 (1.17)	.20**/**	.08	.04
Affiliation: Group	4.86 (1.31)	.02	−.03	.28**/**
Affiliation: Friends	5.30 (1.21)	.04	−.03	.27**/**
Affiliation: Exclusion concern	5.13 (1.31)	−.03	.03	.28**/**
Status-seeking	4.99 (1.35)	−.01	−.03	.23**/**
Mate-seeking	4.18 (1.91)	−.10*/*ns*	−.10*/*ns*	.19**/**
Mate retention	5.47 (1.42)	.13**/*	.10*/*ns*	.15**/**
Kin care: Family members	5.51 (1.10)	.07	.10*/*ns*	.17**/**
Kin care related to children	5.02 (1.59)	.22**/**	< .01	.14**/*
*Gender role stress*				
Physical inadequacy	4.46 (1.16)	.04	.08	.21**/**
Emotional inexpressiveness	3.72 (1.27)	−.02	.03	.10
Subordination to women	2.99 (1.49)	< .01	.07	.17**/**
Intellectual inferiority	3.61 (1.39)	−.01	.03	.15**/**
Performance failure	4.50 (1.33)	.03	.03	.11*/*
*Intrasexual competition*				
Envy	3.23 (1.31)	−.11*/*ns*	< .01	.27**/**
Jealousy	2.97 (1.34)	−.01	.06	.19**/**
Superiority	4.06 (1.28)	−.07	.06	.20**/**

Note: Absolute range for all variables, 1-7

*ns* = Non-significant. *p-*values adjusted using Benjamini-Hochberg’s correction are marked after slashes

*p < .05, ** *p* < .01

Additionally, we used ANOVA to investigate the linearity of the associations between amount of facial hair and other variables. Subordination to women differed depending on amount of facial hair (*F*[3, 410] = 3.07, *p* = 0.03; η^2^ = 0.02); however, Tukey’s post-hoc test did not reveal any significant between-group differences. Envy also differed depending on the amount of facial hair (*F*[3, 410] = 2.72, *p* = 0.04; η^2^ = 0.02); Tukey’s *post-hoc* tests showed lower envy among men with heavy stubble (*M* = 3.03, *SD* = 1.36) compared to men with light stubble (*M* = 3.53, *SD* = 1.31; *t* = 2.65, *p* = 0.04). Inspection of group means did not indicate any non-linear trends. Facial hair enhancement motivation differed between men with different amount of facial hair (*F*[3, 410] = 10.10, *p* < 0.01, η^2^ = 0.07). Clean-shaven men reported lower motivation to enhance facial hair (*M* = 3.79, *SD* = 1.75) compared to men with light stubble (*M* = 4.32, *SD* = 1.42, *p* = 0.04) and with heavy stubble (*M* = 4.44, *SD* = 1.34, *p* < 0.01) who did not differ (*p* = 0.96), but reported lower facial hair enhancement than men with full beard (*M* = 5.39, *SD* = 1.17, *p* < 0.04). We ensured that other association were linear by inspection of scatterplots.

Then we tested whether the amount of facial hair moderates the associations between fundamental social motives, gender role stress, intrasexual competition, and facial hair enhancement motivation. As the moderator is categorical, categories of facial hair were dummy coded with clean-shaven face as the reference group, to examine differences in prediction across three categories of the moderator (men with heavy stubble and full beards were pooled because of low number of participants with full beards [*n* = 23] to ensure more power to detect associations).

**Fig. 2:**
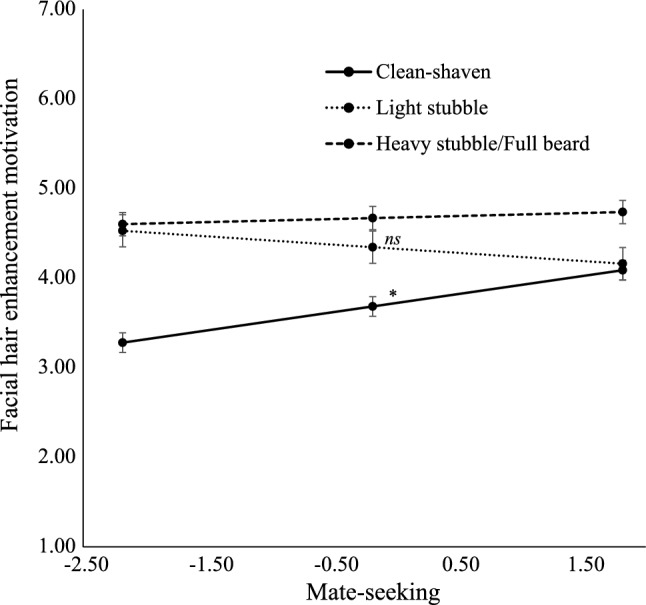
Interaction of mate-seeking motivation and amount of facial hair predicting facial hair enhancement motivation. *Note*. *ns* = Non-significant. Simple slopes analysis showed that mate-seeking motive was associated with greater motivation to enhance one’s facial hair among clean-shaven men compared to men who report other amounts of facial hair. * *p* < .01

A hierarchical regression model with fundamental social motives predicted 19% of variation in facial hair enhancement motivations (*F*[14, 399] = 6.67, *p* < 0.01). Facial hair enhancement motivation was higher among men with high affiliation to friends motive (β = 0.23; *b* = 0.18, *SE* = 0.09; *t* = 2.02, *p* = 0.04) and mate-seeking motive (β = 0.20; *b* = 0.20, *SE* = 0.06; *t* = 3.63, *p* < 0.01). We observed differences in facial hair enhancement motivation between men with light stubble and men with other amounts of facial hair (β = 0.06, *b* = 0.61, *SE* = 0.19; *t* = 3.17, *p* < 0.01) and between men with heavy stubble or full beard and other participants (β = 0.11, *b* = 0.96, *SE* = 0.17; *t* = 5.61, *p* < 0.01). The interaction between amount of facial hair and mate-seeking motive predicted facial hair enhancement motivation (Δ*R*^2^ = 0.02, *F*[2, 399] = 4.34, *p* = 0.01). We observed interactions between the comparison of men with light stubble versus men with other amount of facial hair by mate-seeking motive (*b* = -0.29, *SE* = 0.11; *t* = -2.73, *p* = 0.01), and between the comparison of men with heavy stubble or full beard and other amount of facial hair (*b* = -0.17, *SE* = 0.09; *t* = -1.95, *p* = 0.05). Simple slopes analysis showed that mate-seeking motive was associated with greater motivation to enhance one’s facial hair among clean-shaven men (β = 0.26, *b* = 0.20, *SE* = 0.06; *t* = 3.54, *p* < 0.01) compared to men who report other amounts of facial hair (βs <|0.16|, *bs* <|0.10|, *SE*s = 0.07 to 0.09, *p*s > 0.32; Fig. 2). No other fundamental social motive predicted facial hair enhancement or interacted with the amount of facial hair in predicting facial hair enhancement motivation.

A hierarchical regression model with gender role stress dimensions predicted 12% of variation in facial hair enhancement motivations (*F*[9, 404] = 6.05, *p* < 0.01). Facial hair enhancement motivation was higher among men with high subordination to women (β = 0.29, *b* = 0.27, *SE* = 0.09; *t* = 2.99, *p* < 0.01) and physical inadequacy (β = 0.19, *b* = 0.26, *SE* = 0.08; *t* = 3.29, *p* < 0.01). We observed differences in facial hair enhancement motivation between men with light stubble and other amounts of facial hair (β = 0.05, *b* = 0.48, *SE* = 0.20; *t* = 2.37, *p* = 0.02) and between men with heavy stubble or full beard and other participants (β = 0.08, *b* = 0.75, *SE* = 0.12; *t* = 4.28, *p* < 0.01). Amount of facial hair did not interact with gender role stress dimensions in prediction of facial hair enhancement motivation.

In the hierarchical regression model with dimensions of intrasexual competition, an assumption of non-collinearity was violated (VIF > 10), thus we entered only the sum score of all three dimensions. The modified model predicted 12% of variation in facial hair enhancement motivations (*F*[5, 408] = 11.61, *p* < 0.01). Men who were more intrasexually competitive reported higher facial hair enhancement motivation (β = 0.43, *b* = 0.52, *SE* = 0.10; *t* = 5.15, *p* < 0.01); however, amount of facial hair did not interact with intrasexual competitiveness (Δ*R*^2^ < 0.01, *F* = 2.26, *p* = 0.10).

We found weak support for the hypothesis that facial hair amount moderates the associations between facial hair enhancement motivation and fundamental social motives, gender role stress, and intrasexual rivalry. Thus, we used Steiger’s *z*s to investigate the differences in dependent correlations in a pooled sample of all participants (see Table 1; all Steiger’s z comparisons between correlation coefficients of facial hair enhancement motivation and other variables in a full sample are given in Table S2 in Supplementary online material). The associations between facial hair enhancement motivation and self-protection (*r* = 0.08, *p* = 0.10) and disease avoidance (*r* = 0.04, *p* = 0.42) were weaker compared to the correlations between facial hair enhancement motivation and affiliative motivations and status-seeking (*r*s from 0.23 to 0.28, *p* < 0.01, Steiger’s *z*s ≤ -2.54, *p* < 0.01), physical inadequacy as a dimension of gender role stress (*r* = 0.21, *p* < 0.01, *z* = -2.07, *p* < 0.04), and envy as a dimension of intrasexual competition (*r* = 0.27, *p* < 0.01, *z* = -2.82, *p* < 0.01). We observed stronger associations between affiliation motives and facial hair enhancement compared to the association between mate retention and facial hair enhancement (*z*s ≥ 2.22, *p* < 0.03), and between kin care related to children and facial hair enhancement (*z*s ≥ 2.00, *p* < 0.05). We found that associations between affiliation motives, status-seeking motive and facial hair enhancement motivation were stronger compared to the associations between such dimensions of gender role stress as intellectual inferiority, performance failure, and emotional inexpressiveness (*z*s > 1.93, *p* < 0.05). The differences in the correlations for self-protection and disease avoidance remained after reducing Type 1 error inflation (*z*s < -3.48, adjusted *p* < 0.04).

## Discussion

In our study, facial hair enhancement motivation was associated with amount of facial hair but also correlated with fundamental social motives of affiliation and social status and intrasexual competition. Displaying, augmenting, and enhancing appearance are widespread strategies in intersexual attraction and intrasexual competition (Davis & Arnocky, 2022c; Kowal et al., 2022). Studying the amount of facial hair worn by men and their facial hair enhancement motivation makes it possible to understand the social functions attributed by men to facial hair. To our knowledge, this is the first study that examined the motivation behind facial hair enhancement in men. We demonstrated that facial hair enhancement motivation was associated with the amount of facial hair. Moreover, motivation to care for facial hair was mainly related to affiliation and status motives, but also to intrasexual competition; results were weaker in confirming the precarious masculinity hypothesis (Vandello et al., 2008).

Participants with more facial hair were more oriented toward self-protection. This result is in line with hypotheses about the physical protective function of beards in combat (Beseris et al., 2020) and with hypotheses regarding having a beard as deterring signal for other men (Arnocky & Carré, 2016). Although corresponding to previous findings, this association was relatively weak in our sample and deemed non-significant after correction for the false discovery rate. Thus, future studies should ensure higher statistical power to investigate the association between facial hair and self-protection motive. Moreover, we detected that older men have more facial hair than younger men, which corresponds to previous studies showing that people perceive men with more facial hair as older (Mefodeva et al., 2020). Regarding fundamental social motives, men having more facial hair reported less mate-seeking motivation, but more mate-retention and kin care motivation. These associations remained stable after correcting for multiple comparisons. Previous findings indicated that observers tend to perceive bearded men as having more parenting skills (Dixson et al., 2019). Therefore, having more facial hair may be used by men to inform other people that their social motives shift from focusing on mating market to focusing on long-term romantic relationships and family. In addition, we found that among clean-shaven men mate-seeking motive was linked with a higher motivation to enhance their facial hair, which further supports this thesis. From a different perspective, men reporting more competitiveness did not display more facial hair. This result might indicate that displaying intrasexual rivalry is not the primary function of having facial hair (Dixson & Vasey, 2012). Moreover, gender role stress was not higher among men with more facial hair; therefore, growing facial hair should not be regarded as a compensatory strategy for men unsure of their own masculinity (Vandello et al., 2008). A lack of support for both intrasexual competition and socio-cultural hypotheses might stem from the fact that the effectiveness of biological or social displays depends on their quality. Costly signaling theory (Bliege Bird & Smith, 2005) proposes that humans may send honest signals about having desirable personal characteristics and possessing resources through costly biological displays that would be hard to fake (McAndrew, 2019). Although there is very little evidence that men’s beards are immunologically “costly” and that they advertise superior immune function (Davis & Arnocky, 2022b), in today’s world facial hair can be assessed in aesthetic terms and maintaining aesthetically pleasing facial hair requires costs associated with time and effort. Thus, growing bushy, unhealthy-looking, or dispersed facial hair may discredit a man rather than give him an advantage in intrasexual competition. On the other hand, intrasexual competition among men has two modes, specifically male-male aggression vs. competing for female mate choice (Kordsmeyer et al., 2018; Puts, 2010) and women do not show clear preferences towards men’s faces with facial hair (McIntosh et al., 2017 [supportive results]; Dixson et al., 2013 [non-supportive results]). Because the tool we used to measure intrasexual competition contains several items about the subjects’ relative attractiveness, no items specifically about aggression and contest competition, and several items that are ambiguous regarding mechanism, it may be not sensitive enough to test the prediction that men more often wear beards to enhance their threat potential among men.

We showed that men with higher affiliative and status-seeking motives and more oriented towards intrasexual competition would likely devote more time and resources to care for their facial hair. People perceive bearded men as more trustworthy and friendly (Bakmazian, 2014; Craig et al., 2019). Thus, facial hair enhancement motivations could reflect an orientation to build safe social coalitions based on social status. The mixture of affiliative and competitive motivations which correlated with facial hair enhancement suggests that men who are motivated for facial hair enhancement also want to present themselves as valuable and reliable allies and friends.

We also found that men’s care of their facial looks was associated with socio-cultural motives. Facial hair is a sexually dimorphic ornament that may be important for self-perceived masculinity (McCabe & Riciardelli, 2004). Men experiencing physical inadequacies or concerns of being subordinated to women reported higher facial hair enhancement motivation which might reflect compensatory reaction to threats of their uncertain masculinity (Vandello et al., 2008). However, the sociocultural drivers of facial hair enhancement were considerably weaker compared to social motives and intrasexual competition.

### Limitations and Conclusions

This study was the first investigating facial hair enhancement motivation. We tested various possible predictors of this motivation, including fundamental social motives, gender role stress, and intrasexual competition which allowed us to test both evolutionary and sociocultural hypotheses regarding motivations to care for facial hair. We also recruited a large group of men with different amounts of facial hair which allowed us to quantify possible moderating role of actual amount of facial hair. However, future studies could address some limitations of the present study. First, the measure of actual facial hair captured only a part of many forms in which men could trim and display their facial hair. Such a narrow range of facial hair types to choose from could result in not fully accurate declarations about the respondents’ actual facial hair (i.e., only 6% of respondents declared having a “full beard”). Future studies could use a broader set of stimuli to help the participants report their facial hair more precisely (Gray et al., 2020). Second, the study relied on self-report measures; however, people’s declarations reflect their conscious beliefs, which may not be identical to their actual motivations (Buss, 1991). Thus, future studies could investigate the associations between social motives, intrasexual competition, and gender role stress using observational or behavioral measures. Future studies could develop our ad hoc measure of facial hair enhancement to capture more general motivation to care for facial appearance (e.g., facial skin after shaving). Third, we used single-item scales to measure fundamental social motives that have had some success in other research (Jonason & Tome, 2019). Although single-item scales give results comparable to multi-item scales (Verster et al., 2021), longer scales with stronger psychometric properties are worth considering in future research. Fourth, we did not control participant’s relationship status and some fundamental social motives may have had different importance for singles and people in romantic relationships. Moreover, the distribution of facial hair types in the sample may not have corresponded to the distribution of facial hair types in the general population of Polish men. Future studies could take into account the proportion of different types of facial hair in the general population at least as a context for interpreting the results, especially because facial hair preferences are frequency dependent (Janif et al., 2014). Last, we distinguished dimensions of intrasexual competition identified by Jonason et al., (2023); however, Albert et al., (2022) applied other dimensions of intrasexual competition, that is superiority enjoyment and inferiority frustration. On the other hand, we performed regression analyses on the overall scores, in line with the original procedure (Buunk & Fisher, 2009).

As the first study on facial hair enhancement motivation in men, we found that facial hair may simultaneously signal higher competitiveness and higher willingness to form coalitions and to affiliate. Functions of facial hair displays seem to refer more to social organization and structure of human groups (Sutcliffe et al., 2012) than to sociocultural stress associated with gender role (Wood, 1986). Therefore, men may invest time, effort, and money in facial hair appearance to enhance their social image.

### Supplementary Information

Below is the link to the electronic supplementary material.

Supplementary file1 (DOCX 39 KB)

Supplementary file2 (CSV 69 KB)

## Data Availability

Data has been included as Supplementary material.
