# Supplementary material for: Motivations to Enhance One’s Facial Hair: Affiliation, Rivalry, and Stress
Source: Arch Sex Behav. 2024 Jun 17;53(8):3229–37. doi: 10.1007/s10508-024-02919-0 (PMC11335829; doi:10.1007/s10508-024-02919-0)
Supplement: Supplementary file 1 — Supplementary file1 (DOCX 39 KB) [file 10508_2024_2919_MOESM1_ESM.docx]

**Supplementary online material**

Table S1

*Correlations between study variables*

| Variable | 1 | 2 | 3 | 4 | 5 | 6 | 7 | 8 | 9 | 10 | 11 | 12 | 13 | 14 | 15 | 16 | 17 |
| --- | --- | --- | --- | --- | --- | --- | --- | --- | --- | --- | --- | --- | --- | --- | --- | --- | --- |
|  |  |  |  |  |  |  |  |  |  |  |  |  |  |  |  |  |  |
| 1. Self-protection |  |  |  |  |  |  |  |  |  |  |  |  |  |  |  |  |  |
| 2. Disease avoidance | .58 |  |  |  |  |  |  |  |  |  |  |  |  |  |  |  |  |
| 3. Affiliation – group | .26 | .22 |  |  |  |  |  |  |  |  |  |  |  |  |  |  |  |
| 4.Affiliation – friends | .20 | .35 | .59 |  |  |  |  |  |  |  |  |  |  |  |  |  |  |
| 5. Affiliation – exclusion concern | .28 | .24 | .63 | .59 |  |  |  |  |  |  |  |  |  |  |  |  |  |
| 6. Status-seeking | .25 | .24 | .53 | .40 | .59 |  |  |  |  |  |  |  |  |  |  |  |  |
| 7. Mate-seeking | .01 | .01 | .31 | .27 | .26 | .21 |  |  |  |  |  |  |  |  |  |  |  |
| 8. Mate retention | .31 | .40 | .24 | .41 | .32 | .08 | .14 |  |  |  |  |  |  |  |  |  |  |
| 9. Kin care – family members | .38 | .41 | .38 | .45 | .41 | .27 | .11 | .17 |  |  |  |  |  |  |  |  |  |
| 10. Kin care related to children | .21 | .25 | .32 | .34 | .23 | .21 | .15 | .16 | .05 |  |  |  |  |  |  |  |  |
| 11. Physical inadequacy | .16 | .19 | .28 | .29 | .29 | .27 | .24 | .34 | .19 | .20 |  |  |  |  |  |  |  |
| 12. Emotional inexpressiveness | -.08 | -.10 | .13 | .02 | .12 | .14 | .12 | .01 | -.05 | -.01 | .36 |  |  |  |  |  |  |
| 13. Subordination to women | -.19 | -.23 | .13 | -.04 | .05 | .11 | .26 | -.13 | -.09 | -.03 | .26 | .58 |  |  |  |  |  |
| 14. Intellectual inferiority | -.03 | -.10 | .16 | .03 | .16 | .15 | .20 | .01 | -.01 | -.01 | .39 | .61 | .68 |  |  |  |  |
| 15. Performance failure | .12 | .13 | .19 | .20 | .26 | .17 | .16 | .23 | .18 | .04 | .54 | 30. | .32 | .58 |  |  |  |
| 16. Envy | .01 | -.10 | .18 | .05 | .21 | .16 | .33 | .01 | -.04 | .02 | .33 | .49 | .61 | .58 | .35 |  |  |
| 17. Jealousy | -.06 | -.16 | .09 | -.06 | .08 | .05 | .25 | -.14 | -.08 | -.02 | .20 | .45 | .64 | .52 | .22 | .78 |  |
| 18. Superiority | .12 | .08 | .23 | .17 | .27 | .28 | .29 | .11 | .09 | .04 | .36 | .22 | .29 | .40 | .47 | .55 | .42 |

*Note*. Correlation coefficients higher than ǀ.10ǀ are significant at *p* < .05, and correlation coefficients higher than ǀ.12ǀ are significant at *p* < .01. Correlation coefficients higher than ǀ.10ǀ and higher that ǀ.13ǀ are significant at *p* < .05 and *p* < .01, respectively, according to Benjamini-Hochberg’s correction adjusted *p*- values.

Table S2

*Steiger’s z comparisons between correlation coefficients of facial hair enhancement motivation and other variables in a full sample*

| Variables | 1 | 2 | 3 | 4 | 5 | 6 | 7 | 8 | 9 | 10 | 11 | 12 | 13 | 14 | 15 | 16 | 17 |
| --- | --- | --- | --- | --- | --- | --- | --- | --- | --- | --- | --- | --- | --- | --- | --- | --- | --- |
| 1. Self-protection |  |  |  |  |  |  |  |  |  |  |  |  |  |  |  |  |  |
| 2. Disease avoidance | 0.89 |  |  |  |  |  |  |  |  |  |  |  |  |  |  |  |  |
| 3. Affiliation – group | **-3.43**/*** | **-4.01**/**** |  |  |  |  |  |  |  |  |  |  |  |  |  |  |  |
| 4.Affiliation – friends | **-3.13**/*ns*** | **-4.20**/**** | 0.24 |  |  |  |  |  |  |  |  |  |  |  |  |  |  |
| 5. Affiliation – exclusion concern | **-3.48**/*** | **-4.06**/**** | < 0.01 | -0.24 |  |  |  |  |  |  |  |  |  |  |  |  |  |
| 6. Status-seeking | **-2.54**/n*s*** | **-3.18**/*ns*** | 1.09 | 0.77 | 1.17 |  |  |  |  |  |  |  |  |  |  |  |  |
| 7. Mate-seeking | -1.61 | **-2.19*/*ns*** | 1.62 | 1.40 | 1.56 | 0.67 |  |  |  |  |  |  |  |  |  |  |  |
| 8. Mate retention | -1.22 | **-2.05*/*ns*** | **2.22*/*ns*** | **2.31*/*ns*** | **2.34*/*ns*** | 1.23 | 0.63 |  |  |  |  |  |  |  |  |  |  |
| 9. Kin care – family members | -1.66 | **-2.45*/*ns*** | **2.08*/*ns*** | **2.00*/*ns*** | **2.13*/*ns*** | 1.04 | 0.31 | -0.32 |  |  |  |  |  |  |  |  |  |
| 10. Kin care related to children | -0.98 | -1.67 | **2.52*/*ns*** | **2.37*/*ns*** | **2.37*/*ns*** | 1.49 | 0.79 | 0.16 | 0.45 |  |  |  |  |  |  |  |  |
| 11. Physical inadequacy | **-2.07*/*ns*** | **-2.75**/*ns*** | 1.24 | 1.06 | 1.24 | 0.35 | -0.34 | -1.08 | -0.65 | -1.15 |  |  |  |  |  |  |  |
| 12. Emotional inexpressiveness | -0.28 | -0.82 | **2.86**/*ns*** | **2.54*/*ns*** | **2.84**/*ns*** | **2.06*/*ns*** | 1.40 | 0.73 | 0.99 | 0.43 | 1.83 |  |  |  |  |  |  |
| 13. Subordination to women | -1.20 | -1.70 | 1.76 | 1.46 | 1.69 | 0.94 | 0.34 | -0.28 | < 0.01 | -0.43 | 0.68 | -1.57 |  |  |  |  |  |
| 14. Intellectual inferiority | -1.00 | -1.52 | **2.11*/*ns*** | 1.81 | **2.11*/*ns*** | 1.28 | 0.66 | < 0.01 | 0.29 | -0.15 | 1.13 | -1.16 | 0.52 |  |  |  |  |
| 15. Performance failure | -0.46 | -1.08 | **2.80**/*ns*** | **2.64**/*ns*** | **2.93**/*ns*** | **1.93*/*ns*** | 1.27 | 0.66 | 0.96 | 0.59 | **2.15*/*ns*** | -0.17 | 1.06 | 0.89 |  |  |  |
| 16. Envy | **-2.82**/*ns*** | **-3.23**/*ns*** | 1.16 | 0.92 | 1.18 | 0.32 | -0.36 | -0.89 | -0.56 | -1.04 | < 0.01 | **-2.25*/*ns*** | -0.94 | -1.36 | -1.81 |  |  |
| 17. Jealousy | -1.56 | **-2.02*/*ns*** | 1.42 | 1.16 | 1.41 | 0.61 | < 0.01 | -0.55 | -0.28 | -0.73 | 0.33 | -1.77 | -0.49 | -0.84 | -1.32 | 0.63 |  |
| 18. Superiority | -1.87 | **-2.43*/*ns*** | 1.36 | 1.14 | 1.40 | 0.52 | -0.18 | -0.78 | -0.46 | -0.90 | 0.18 | -1.65 | -0.52 | -0.95 | -1.80 | 0.22 | -0.19 |

*Note.* *ns* = non-significant. *p-*values adjusted using Benjamini-Hochberg’s correction are marked after slashes.

* *p* < .05, ** *p* < .01
